# Supplementary material for: Cryoprotective Metabolites Are Sourced from Both External Diet and Internal Macromolecular Reserves during Metabolic Reprogramming for Freeze Tolerance in Drosophilid Fly, Chymomyza costata
Source: Metabolites. 2022 Feb 9;12(2):163. doi: 10.3390/metabo12020163 (PMC8877510; doi:10.3390/metabo12020163)
Supplement: Supplementary file 1 [file metabolites-12-00163-s001.zip › Supplementary Figures_Metabolites.pdf]

Supplementary Figures for:

Accumulation of innate cryoprotective metabolites in a freeze-tolerant insect: internal macromolecular reserves or diet as alternative sources of carbon skeletons.

Martin Moos, Jaroslava Korbelová, Robert Grgac, Tomáš Štětina, Stanislav Opekar, Petr Šimek, Vladimír Koštál

Figure S1 PAGE analysis of total proteins.

Figure S2 Fluxomics of ^{13}C-labelled metabolic precursors of betain.

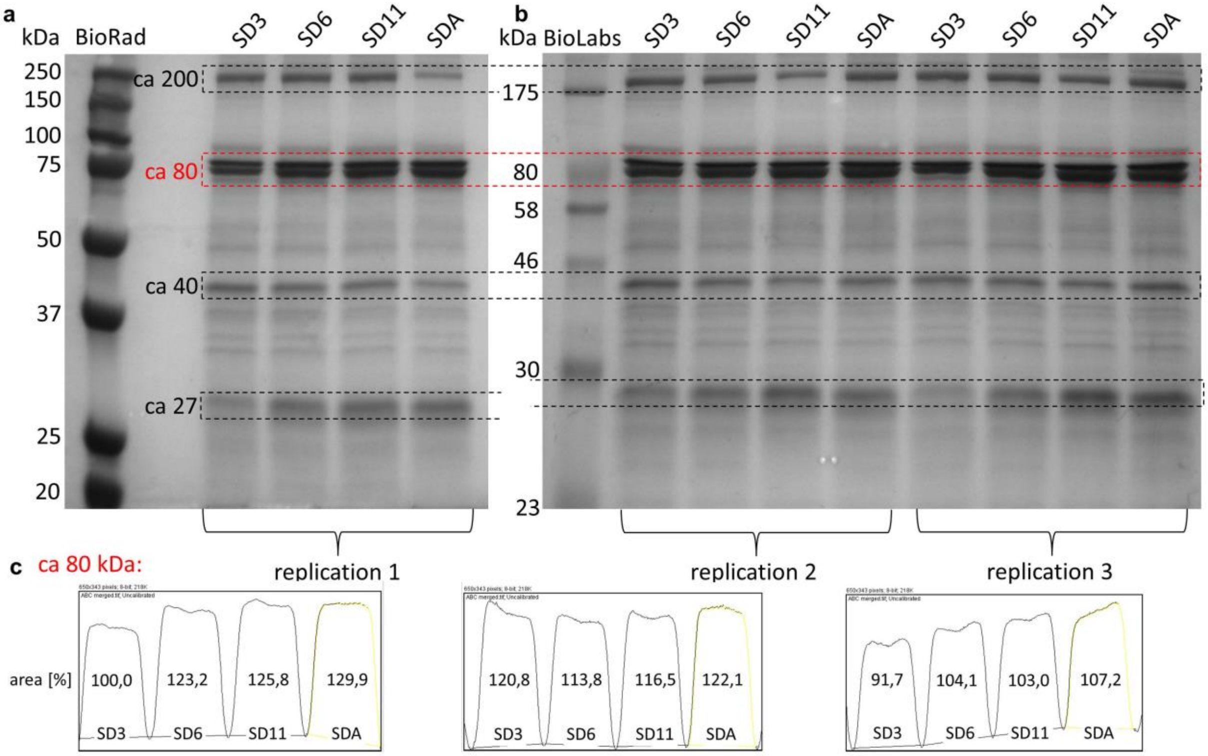

d

	ca 200 kDa		ca 80 kDa		ca 40 kDa		ca 27 kDa	
	mean	s.d.	mean	s.d.	mean	s.d.	mean	s.d.
SD3	90,9	7,7	104,2	12,2	102,2	4,3	91,4	16,3
SD6	84,7	10,7	113,7	7,8	89,1	4,2	106,5	10,2
SD11	68,4	21,7	115,1	9,4	82,3	13,0	118,5	14,2
SDA	72,7	14,1	119,7	9,4	90,2	5,6	119,5	17,1
Anova *F*:	1,546		1,316		3,474		2,392	
Anova *P*:	0,2762		0,3349		0,0706		0,1441	
	ns		ns		ns		ns	

Figure S1: PAGE analysis of total proteins.

Total proteins were extracted from different acclimation variants of larvae of *C. costata* (SD3, SD6, SD11, SDA; for description, see Fig. 8). Protein extracts were loaded onto precast 7.5% MiniProtean TGX gels and separated by polyacrylamide gel electrophoresis (PAGE). Protein bands were stained with Coommassie Brilliant Blue and band densities in selected areas (ca 200, ca 80, ca 40, and ca 27 kDa as delimited by broken rectangles) were compared using ImageJ software. **(a)** First replicate of total protein extracts. BioRad, Precision Plus Protein Dual Color Standards. **(b)** Second and third replicates. BioLabs, ColorPlus Prestained Protein Marker, Broad Range. **(c)** Example of ImageJ analysis for the protein area of ca 80 kDa (red rectangle). Two dense bands are most likely corresponding to Larval serum proteins Lsp1 and Lsp2, which are the most adundant proteins in drosophilid larval hemolymph [1]. The area under density peak of the first band of replication 1 is set to 100% and all other bands are normalized to it. **(d)** Results of statistical analysis using one way Anova (the result for protein area of ca 80 kDa is highlighted in red font). No significant influence of phenotypic variant on density of protein bands was detected (Anova $P > 0.05$; ns, non-significant).

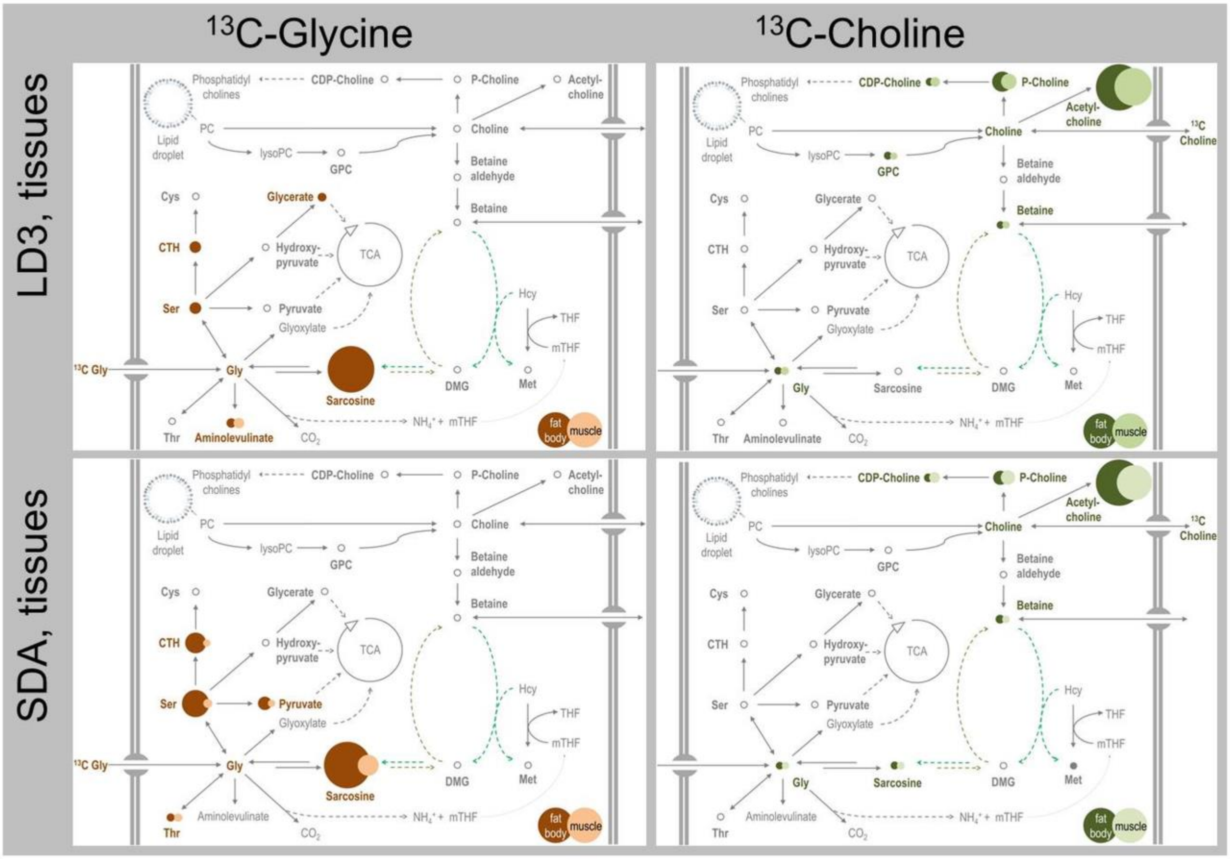

Figure S2: Fluxomics of ^{13}C-labelled metabolic precursors of betain.
The maps show metabolic destiny of two ^{13}C-labelled compounds (^{13}C-glycine, left maps; and 1,2-^{13}C2 choline chloride, right maps) added to the medium (Schneider's Drosophila Medium, Biosera), in which fat body and muscle tissues (distinguised by shade as indicated) dissected from LD (3-w-old, upper row) and SDA (11-w-old, lower row) larvae were incubated for 3 h (LD) or 6 h (SDA) and then processed and analyzed using HRMS metabolomics platform (for more detail, see Materials and Methods). The results are expressed for each metabolite as a percentage of the peak area of ^{13}C-form (subtracting the native occurrence of ^{13}C-carbon) relative to ^{12}C-form (100% is a small circle in the Legend, at 0h). The size of circles is proportional to the percentage of ^{13}C-form. For reference to enzymes (arrows), see Fig. 6.

Note: Fig. S2 brings basically the same general message as Fig. 6: The ^{13}C glycine can be methylated only once, forming sarcosine. Di- and tri-methylation-conversions of glycine to DMG and betaine, respectively, were not observed, which suggests that these conversions, characteristic for microbial halophiles, do not exist in *C. costata* larval tissues. The ^{13}C choline chloride can be converted to betaine and also to sarcosine and glycine, which suggests that this classical animal pathway is operational also in *C. costata* larval tissues.

References in Supporting materials

1. Powell, D.; Sato, J. D.; Brock, H. W.; Roberts, D. B., Regulation of synthesis of the larval serum proteins of Drosophila melanogaster. *Dev. Biol.* **1984,** 102, (1), 206-215.
